# The continuum of carbon–hydrogen (C–H) activation mechanisms and terminology

**DOI:** 10.1038/s42004-021-00611-1

**Published:** 2021-12-10

**Authors:** Kristof M. Altus, Jennifer A. Love

**Affiliations:** 1 Department of Chemistry, The University of British Columbia, Vancouver, BC V6T 1Z1 Canada; 2 Department of Chemistry, University of Calgary, 2500 University Drive N.W., Calgary, AB T2N 1N4 Canada

**Keywords:** Organometallic chemistry, Homogeneous catalysis

## Abstract

As a rapidly growing field across all areas of chemistry, C-H activation/functionalisation is being used to access a wide range of important molecular targets. Of particular interest is the development of a sustainable methodology for alkane functionalisation as a means for reducing hydrocarbon emissions. This Perspective aims to give an outline to the community with respect to commonly used terminology in C-H activation, as well as the mechanisms that are currently understood to operate for (cyclo)alkane activation/functionalisation.

## Abstract (web summary)

The field of C-H activation has boomed in the past two decades, but the diverse and overlapping terms used in the literature can be difficult to parse. Here the authors review key mechanistic concepts and offer a guide to the perplexed.

## Introduction

The replacement of a hydrogen atom in a carbon-hydrogen (C-H) bond by another element or functional group brings together collaborations from all areas of chemistry. The replacement of the hydrogen atom in methane is so important that it has been highlighted as one of the grand challenges of chemistry^1,2^. The interest stems from the high atom economy these transformations provide along with the potential environmental benefits of using alkanes as chemical building blocks. The two main challenges regarding these reactions are (1) poor selectivity, due to the abundance of C-H bonds with similar bond dissociation energies and reactivity, and (2) the chemical inertness of hydrocarbon C-H bonds, often due to the inherent low polarity. Furthermore, the potential atom economy of C-H activation/functionalisation reactions remains limited by the need for stoichiometric reagents, such as oxidants, which are critical in most reactions and hamper uptake by industry.

Notwithstanding these challenges, the field has thrived in the last 20 years, with a considerable number of new methods being developed for the activation and functionalisation of a wide variety of C-H bonds. Several reviews covering reactivity tailored towards complex target molecules have been published^3–11^. Although significantly less well understood, the increasing usage of Earth-abundant metals in catalyst development has not escaped the C-H functionalisation community, and reviews highlighting their importance are available^12–15^. Early transition metals have also proven effective in C-H bond activation and functionalisation^16–18^. Developments towards water-stable reactivity^19^, as well as electrochemical reactions, are also prevalent^20^. Furthermore, a myriad of computational work regarding the breaking of C-H bonds has also been described^21^.

An unanticipated result of the rapid growth of C-H activation strategies has been the inconsistent use of terminology about activation versus functionalisation, as well as mechanisms of activation. This Perspective is intended to build on the 2002 Nature paper by Labinger and Bercaw which delineated different mechanisms of C-H activation in the preceding decades^22^. Whereas Bercaw and Labinger provided a useful resource for references prior to 2002, which remains relevant, here we seek to provide a framework for researchers to understand and communicate clearly about C-H activation mechanisms based on work published in the last 20 years.

This Perspective was prompted by a discussion at the Faraday meeting in 2019 between Love, Eisenstein, Macgregor, Davies, Lloyd-Jones and others^23^. A point of agreement was that the use of terminology to describe and differentiate between the processes involved in C-H bond cleavage within the community was being used inconsistently. Additionally, Lloyd-Jones remarked that conclusions based on kinetic isotope effect (KIE) in many synthetic/catalysis papers were misinterpreted to show that C-H activation is rate-determining when it may not be; we will not be discussing KIEs in this Perspective but in depth reviews by Jones as well as Gómez-Gallego and Sierra who cover the topic in close detail are available^24,25^. For another thorough discussion of large KIEs in organometallic chemistry, see the minireview by Miriam Bowring^26^.

Since Labinger and Bercaw reviewed the field, there have been significant developments in C-H activation, including new mechanisms (e.g. amphiphilic metal-ligand activation (AMLA) and concerted metalation deprotonation (CMD), Box 1), breakthroughs in the isolation of σ_C-H_ complexes, catalytically relevant examples of hydrocarbon functionalisation, and an expanded understanding of these reactions and their mechanisms. Based on the work of many researchers outlined in the text below, we argue that C-H activation should not be categorised based on metal/ligand combinations, but rather the degree of net charge transfer between the fragments involved in the transition state (electrophilic, amphiphilic, nucleophilic, see below). We outline the mechanisms that are currently understood to operate for (cyclo)alkane activation and some of the terminology commonly used in the C-H activation community.

Box  1  List  of  acronyms KIE – Kinetic Isotope Effect

**Electrophilic mechanisms**

ES – Electrophilic Substitution

AMLA – Ambiphilic Metal-Ligand Activation

CMD – Concerted Metallation Deprotonation

eCMD – Electrophilic Concerted Metallation Deprotonation

LLHT – Ligand to Ligand Hydrogen Transfer

**Oxidative mechanism**

OA – Oxidative Addition

**Sigma bond metathesis mechanisms**

SBM – Sigma Bond Metathesis

OHM – Oxidative Hydrogen Migration

OATS – Oxidatively Added Transition State

σ-CAM – Sigma Complex Assisted Metathesis

MAσBM – Metal-Assisted Sigma Bond metathesis

## Scope

We outline the mechanisms that are currently understood to operate for (cyclo)alkane activation and some of the terminology commonly used in the C-H activation community to bring clarity to a field that is undergoing rapid growth.

While C-H bond reactivity covers a large variety of C-H bonds with differing BDEs (bond dissociation energy), we will focus on processes involved in breaking C-H bonds of alkanes and cycloalkanes and the nuances that govern their reactivity. We will discuss the major differences in the mechanisms of C-H activation. These can be classified as amphiphilic metal-ligand activation or concerted metallation deprotonation (AMLA or CMD), oxidative addition (OA), sigma bond metathesis and 1,2-addition. We will also discuss the use of terminology, such as the differences between functionalisation and activation as well as sigma vs agostic interactions. We will use the classical representations of the mechanisms to break down the transition states involved in each mechanism, to maintain clarity and ease of understanding. Although it is important to realise that a continuum of reactivity is a more accurate descriptor of how to categorise the mechanisms, it is not the most intuitive, especially without the use of computational methods.

**Fig. 1:**
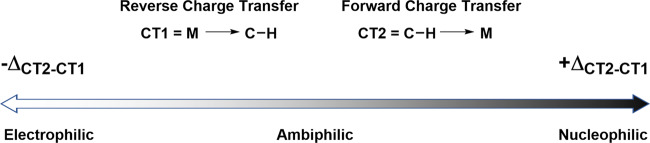
Continuum of charge transfer. Representation of the continuum of charge transfer in C-H activation reaction pathways.

Ess, Goddard and Periana challenged the conventional classification of C-H cleavage mechanisms in a computational study^27^. The authors deconstructed the classical perception that C-H cleavage reactions are segregated into specific mechanisms based on the type of metal (i.e. late or early), the ligands, and the number of atoms involved in the transition state. They provided a detailed energy decomposition analysis of the transition states and reaction pathways involved in C-H activation reactions. This showed that the factor dictating the mechanisms is the overall degree of charge transfer from a metal d_π_-orbital to the C-H σ*-orbital (CT1, reverse charge transfer, Fig. 1) and the charge transfer from the C-H σ-orbital to a metal d_σ_-orbital (CT2, forward charge transfer, Fig. 1). A key conclusion is that instead of segregated mechanisms, a continuum of reactivity exists ranging from electrophilic, through amphiphilic to nucleophilic in character. The traditional mechanisms can therefore be categorised on this scale by the overall difference in charge transfer during the transition state and not, for example, the overall charge of the complex or formal oxidation state of the metal involved. This concept had loosely been applied by Cundari, Macgregor and Davies some years prior (Fig. 1)^28,29^. We recommend this paper to gain a clear understanding of this concept with examples of a diverse range of transition states that exhibit different positions on the continuum.

## C-H activation vs functionalisation

Terminology is an important part of chemistry and defines the way that chemists converse with each other effectively. Within the field of C-H bond cleavage, both the terms 'activation' and 'functionalisation' are becoming increasingly popular in the literature, and in some cases are being used inconsistently. We feel it is important to distinguish the two and offer up definitions that are in line with usage in the wider organometallic community. We acknowledge that different definitions of these terms may exist, but we use these in this Perspective for consistency

### C-H activation

A specific mechanistic step involving the direct cleavage of a C-H bond that occurs due to an interaction with a transition metal, where the result is a new carbon-metal bond.

### C-H functionalisation

A process involving the replacement of a C-H bond by another element or functional group but where the functionalisation is most often preceded by a C-H activation event.

It is also noteworthy that in the context of breaking a C-H bond the term 'activation' should not refer to the elongation or change in polarity of a C-H bond upon coordination to a transition metal. This could be misconstrued, as the word 'activation' implies that the C-H bond is in an altered state. While this statement is true, the new carbon-metal fragment that is generated upon cleavage can also be described as an 'activated' state. It is this definition that has become the accepted convention within the organometallic field.

## Sigma and agostic complexes

Sigma and agostic interactions have been established as the principal step prior to C-H bond activation^30^. It must be recognised that in almost all cases these interactions are crucial for activating a C-H bond by stabilising high energy metal intermediates and polarising the C-H bond to allow for cleavage to occur. Both terms describe the same interaction: the donation of electron density from the σ-orbital of a C-H bond into an empty d-orbital on a transition metal. The difference, however, is in the connectivity of the molecule undergoing the interaction. Sigma complexes are classified as C-H bonds undergoing this interaction through an intermolecular approach. An agostic complex, a term coined by Brookhart and Green^31^, is therefore classified as an intramolecular approach by a C-H bond that is held in the coordination sphere of the metal due to another primary metal–ligand interaction^32,33^.

**Fig. 2:**
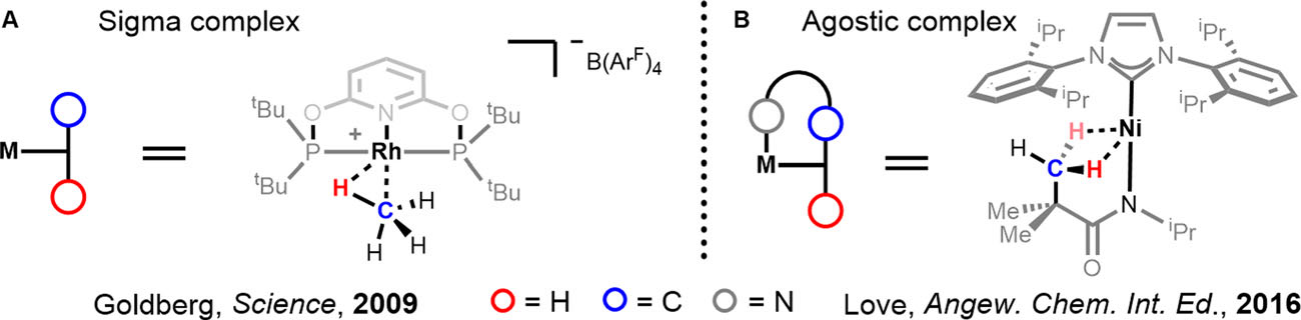
Examples of sigma and agostic complexes. **A** First methane sigma complex fully characterised in situ. **B** Agostic complex featuring two agostic interactions.

Sigma interactions are weak and as such these complexes are generally not isolable. However, time-resolved infrared spectroscopy (TR-IR) has been used to show the existence of such species^34–36^. In 2009 the first methane sigma complex was fully characterised in solution by Goldberg and co-workers in what was then an extremely rare example (Fig. 2A)^37^. Multiple sigma and agostic interactions have also been reported, notably by Ball and co-workers^38^. one example of a multiple agostic interaction is the nickel complex shown by Beattie and co-workers (Fig. 2B)^39^. Further reports of methane and longer (cyclo)alkanes forming sigma complexes have since been shown and supported with kinetic, computational and X-ray experiments^38,40–43^. For a detailed collection on the characterisation of sigma complexes, see the minireview by Young^30^.

## The mechanisms

C-H activation processes have historically been separated into three or four different classical mechanisms. Each mechanism can be described by a variety of factors such as whether the metal is an early or late transition metal, whether the oxidation state of the metal changes if a ligand is involved during the transition state of the bond cleaving event, and or the type of ligand involved. As discussed above, the notion of separating the mechanisms is changing as more and more examples are developed that contradict these descriptors as highlighted by Ess et al.^27^.

The mechanistic nuances that we will cover are based on activation of alkanes and will be grouped according to the historical four categories: electrophilic, oxidative addition, sigma bond metathesis and 1,2-addition mechanisms. We have chosen to keep the mechanisms grouped under these historical categories for clarity and to maintain focus on the steps involved in the breaking of the C-H bond in each respective mechanism.

The electrophilic mechanisms, namely substitution/CMD/AMLA (see Box 1 for a list of acronyms), generally occur at electropositive late transition metal complexes. In these systems, the oxidation state of the metal does not change during the C-H activation step. Typically, carboxylate ligands are used as an intramolecular base to deprotonate the alkane.

Oxidative addition, one of the most studied fundamental transformations in organometallic chemistry, is generally reserved for low valent electron-rich metal complexes featuring strongly donating L-type (L = neutral) ligands, such as those often seen in dehydrogenation chemistry. In this mechanism the C-H bond breaks, increasing the metal’s oxidation state and coordination number by two.

Sigma bond metathesis (SBM) generally involves the early transition metals when d electrons are not available for oxidative addition. A four-centred transition state is operative in which the H atom from a C-H bond is transferred to an existing M-C bond. The H atom acceptor normally dissociates from the metal complex upon acceptance of the H atom. In sigma bond metathesis systems, the oxidation state does not change throughout the reaction. The receiving ligand is generally another hydrocarbyl or hydride fragment, but can also be a main group substituent such a boryl or silyl.

1,2-Addition across a multiple bond is generally associated with early transition metals. In this mechanism, the hydrogen atom from the C-H fragment adds across a double or triple bond, thereby reducing the atom bound to the metal and forming a new M-C bond in the process. These reactions are most often seen in metals such as Zr and Ti, featuring multiply bonded carbon or heteroatoms.

In the following section, we will review a set of examples from each class of mechanism. We will then discuss some of the important key concepts that underpin each type of mechanism.

## Electrophilic (ES and AMLA/CMD)

The electrophilic mechanism of C-H activation first came to light with the pioneering work of Shilov, which was later expanded upon by Bercaw, Labinger and many others^44–46^. The guiding requirement for a C-H activation to be considered as an electrophilic mechanism is the abstraction of a proton by an available lone pair on the neighbouring heteroatom during the transition state. This key feature also distinguishes the electrophilic substitution (ES) mechanism from the sigma bond metathesis mechanisms, which also have a four-centred transition state but no lone pair involvement. In these systems, the oxidation state of the metal normally doesn’t change and remains constant throughout the process. Generally, the formation of a ring that includes the metal, the C-H fragment and the heteroatom is present. However, examples without ring formation have also been calculated as possible pathways^47^.

**Fig. 3:**
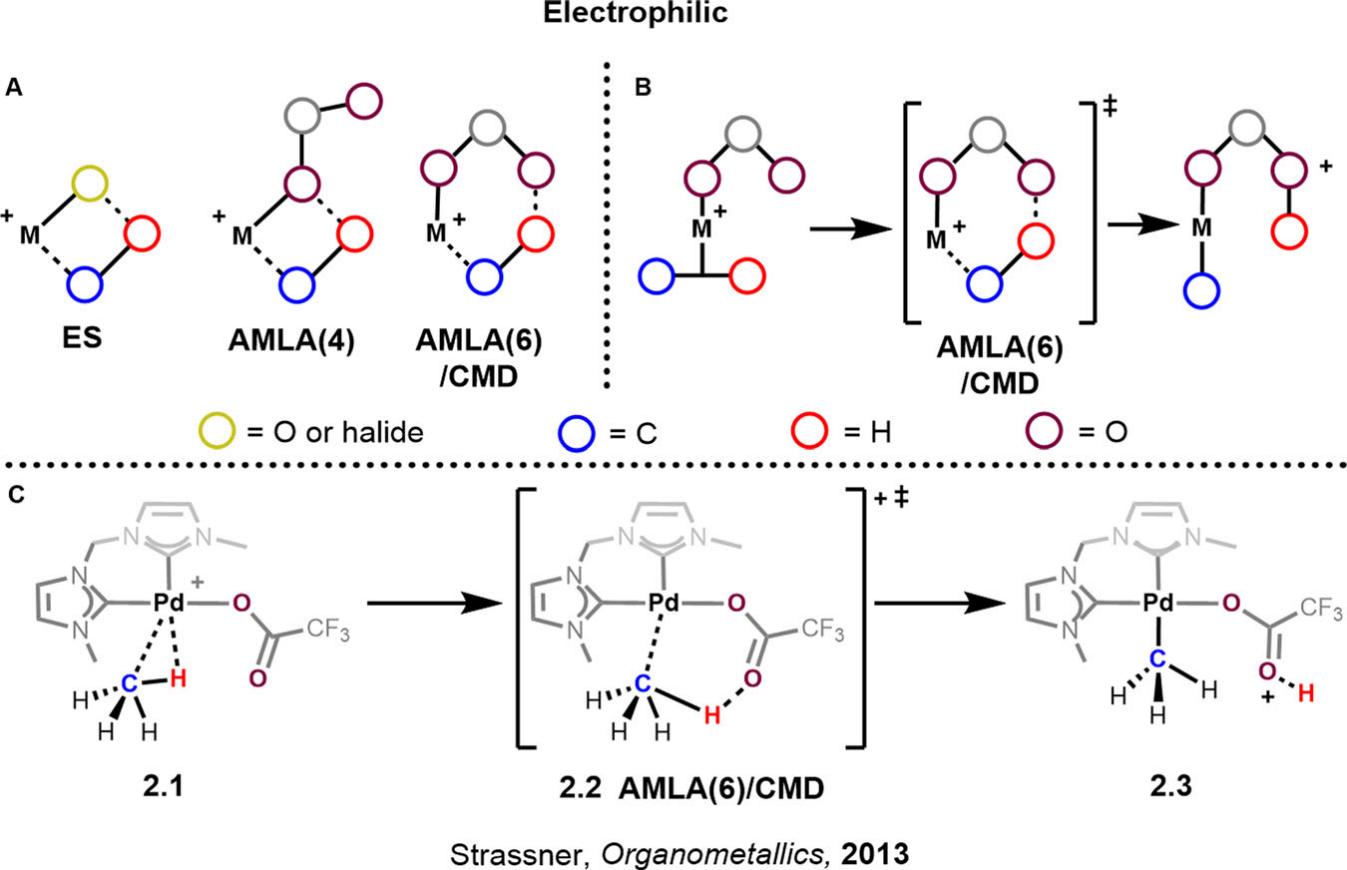
Electrophilic mechanisms. **A** Three most common transition states are found in electrophilic C-H activation. **B** Simplified depiction of the AMLA(6)/CMD mechanism. **C** DFT calculated mechanism for the C-H activation of methane at (NHC)_2_Pd(II) via the AMLA(6)/CMD transition state.

There are currently a number of classifications for (cyclo)alkanes (Fig. 3A) which were reviewed by Goddard, Ess and Periana^48^. Another classification reserved for electron-deficient aromatic systems is known as eCMD (electrophilic concerted metallation deprotonation), a term coined by Carrow^49^. In 2012, a new mechanism was proposed by Perutz and Eisenstein to be operative in first row metals, known as a ligand to ligand hydrogen transfer (LLHT)^50^. Electrophilic substitution has been the topic of many reviews and detailed studies^44^. We will therefore turn our attention towards AMLA/CMD and the recent advances therein.

Arguably the most important subfield of C-H activation for transformations related to pharmaceutical and agrochemical molecules are AMLA, coined by Macgregor and Davis^51^, and CMD, coined by Fagnou^52^. Both terms describe the base-assisted cleavage of a C-H bond, and have become increasingly useful in the quest for a homogenous catalytic process for the activation of alkanes. During AMLA/CMD, a late transition metal (Ir, Pd, Pt, etc) forms a sigma bond with an alkane (Fig. 3C, **2.1**). The formation of the sigma complex polarises the C-H bond, thereby increasing the acidity of the proton. This allows for a weak base, classically a carboxylate ligand, to deprotonate the hydrocarbon leading to a new metal-carbon bond and a carboxylic acid (Fig. 3C, **2.2** **→ 2.3**).

Computational calculations have been instrumental in teasing apart the smallest of details regarding C-H activation. For example, while a significant proportion of complexes studied for AMLA/CMD have been some variation of an electropositive Ir(III) metal complex, many of the transition states have been assigned as amphiphilic in character (to be both electrophilic and nucleophilic). This seems counterintuitive, but the net transfer of electrons between the metal, the internal base and the C-H bond is small, and it is this balance of charge density that has resulted in the term AMLA being assigned to this process.

Further calculations have also shown that the energy associated with distorting the geometry of the metal complex upon the approach of the C-H bond (distortion energy) dictates whether the AMLA(4) or AMLA(6) transition state will be favourable^48^. Another factor dictating the mechanism is the M-H interaction: as it becomes more pronounced, the transition state becomes increasingly more oxidative in character, showing the continuum between the electrophilic and oxidative addition mechanisms^29^.

Consequently, the main difference between the AMLA and CMD mechanisms refers to where they fall on the continuum with respect to each other. AMLA can be viewed as more amphiphilic whereas CMD is more nucleophilic or oxidative. Another important difference lies in the number of transition states with AMLA processes possessing two transition states and CMD only one. A review by Macgregor and Davies explores some of these differences^53^.

**Fig. 4:**
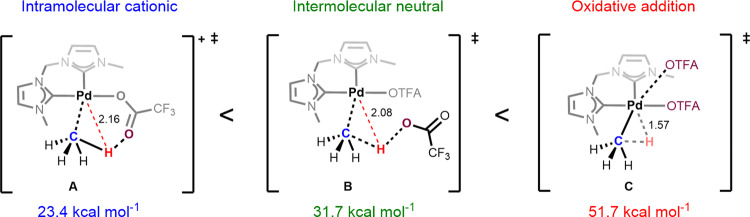
Calculated transition states for the C-H activation of methane at Pd(II). All energies are relative to sigma complex **2.1**, Fig. 2. Pd-H red dashed lines in **A** and **B** do not represent an interaction, instead are representative of distance to the metal, whereas the dashed line in **C** does represent a Pd-H interaction.

As more details are revealed by computational studies, the importance of ligand design becomes increasingly more relevant. With the understanding of charge transfer and geometrical constraints within the transition state, more elaborate and finely tuned ligands can be designed that address potential shortcomings. For instance, the Strassner group has for the better part of two decades been describing the activation of methane and other small alkanes by employing a chelating bis-*N*-heterocyclic carbene palladium complex that operates under the AMLA/CMD mechanism (Fig. 3C, **2.1**)^54–56^. Considering the classical descriptions for C-H activation reactions, one could expect this complex to be susceptible to oxidative addition due to the strongly donating carbenes which impart considerable sigma donation with very little to no back donation increasing the electron density on the metal^57,58^. However, the oxidative addition pathway was calculated to be 28.3 kcal/mol higher in energy than the AMLA(6) mechanism (AMLA(4) not calculated). A third transition state involves the C-H bond being deprotonated by a trifluoracetato group in an intermolecular fashion; the authors describe this transition state as intermolecular neutral and it was calculated to be 8.3 kcal/mol higher in energy than the AMLA(6) mechanism (Fig. 4). Interestingly, the three calculated transition states further illustrate the mechanistic continuum. We can see from the calculated bond distances that as we move between the transition states and as they become more oxidative in character, the M-H distance gets smaller until an M-H bond is formed (2.16 > 2.08 > 1.57 Å). Similarly, the O-H interaction between the carboxylate oxygen and the C-H bond also decreases towards the oxidative addition transition state until the interaction becomes negligible. Charge transfer stabilisation energies have not been calculated on these transition states and can therefore not be given a specific position on the continuum.

Although the differences in energy between these transition states are large and indicate a preference for the AMLA/CMD mechanism, caution must be used when assigning a mechanism to a process that has not undergone extensive kinetic and or computational modelling. From this example alone we can see the diversity of potential mechanisms at play and these three mechanisms could easily be closer in energy with modified ligands or different metals.

Further developments from the Strassner group have included the activation and functionalisation of propanes to a variety of products which was hampered by poor selectivity^59,60^. Ligands containing charge-shift bonding may provide improvements in the activity for C-H activation of alkanes as shown in calculations by Ma and Zhang^61^. The charge-shift bonding motif in the 2-borabicyclo [1.1.0] but-1(3)-ene ligand scaffold imparts more electron density onto the palladium centre compared to the bis-NHC scaffold and results in a smaller M-C contact during the transition state which in turn lowers the barrier to the terminal C-H activation in propane.

The electrophilic mechanisms, especially AMLA/CMD, have been shown to be involved in many C-H activation processes. Active efforts in this area continue and may lead to an industrial process capable of functionalising methane and higher-order alkanes in an efficient manner.

## Oxidative addition (OA)

Oxidative addition is one of the most studied fundamental mechanistic steps in inorganic chemistry. In this mechanism, the formal oxidation state and the coordination number of the metal is increased by two units. It has long been believed that oxidative addition reactions are exclusive to electron-rich late transition metals. However, that notion has changed significantly over the last 20 years. Examples of oxidative transition states and intermediates have been calculated for cationic and electron-deficient complexes such as those in Shilov type systems^46,62^. A recent study, following up on calculations initially carried out by Cundari has shown that strong sigma donor ligands trans to the site of activation increase the barrier to oxidative addition of alkanes versus weakly sigma donating and strongly pi donating ligands^28,63^. It has also been shown that the oxidative addition mechanism is closely linked to that of the sigma bond metathesis mechanism, a mechanism that has been considered to occur exclusively in early transition metals. The findings highlight that the alkyl hydride intermediate for oxidative addition mechanisms can serve as a transition state during some sigma bond metathesis reaction pathways where a temporary M-H (oxidative addition) interaction exists.

**Fig. 5:**
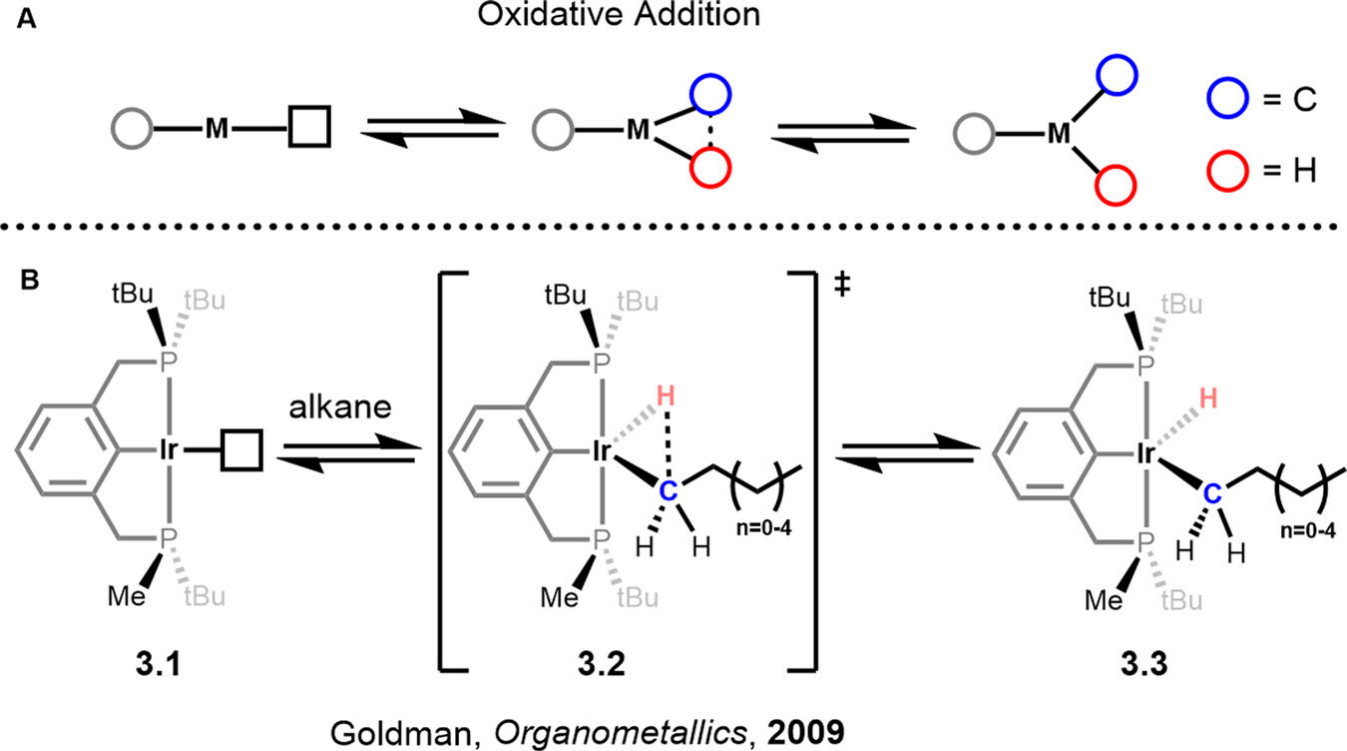
Oxidative addition mechanism. **A** Simplified depiction of the oxidative addition mechanism. **B** Concerted oxidative addition of an alkane to an unsaturated Iridium(I) PCP complex.

The concerted mechanism shown in Fig. 5 (TS **3.2**) is the most common pathway for the activation of C-H bonds via oxidative addition. The development of the PCP/POCOP pincer ligands have revolutionised the dehydrogenation chemistry of alkanes and many groups have contributed to this field. Excellent reviews on the intricacies of ligand design and catalytic methods are available^64,65^. An example of this dependency can be seen in the chemistry reported by the Goldman group, which shows that steric control enables the fine-tuning of energetic barriers to C-H activation^66^. Reduction of the steric environment in the active site lowers the activation barrier to C-H activation. However, insufficient steric crowding can lead to multinuclear cluster formation, inhibiting catalysis. The C-H activation barrier was lowered by about 5 kcal/mol when a single tert-butyl group was replaced with a methyl group. The bulky tert-butyl groups on the phosphines are important for creating an open coordination site trans to the phenyl position allowing for alkane coordination and activation (**3.3**, Fig. 5). The alkane can then orientate itself in one of two directions, towards the methyl substituent or towards the tert-butyl with the former being 3 kcal/mol lower in energy. Subsequent substitution of the tert-butyl groups for methyl substituents does not lower the activation barrier any further but could increase the amount of multinuclear cluster formation. This effect is a reminder that simply looking at the two extremes, be it for sterics or electronics may not always give the full answer and that vital information about a transition state could be lost.

The transition metal complexes of carbon monoxide (CO) have become crucial in understanding oxidative addition reactions of C-H bonds. Photolysis can be used to readily dissociate a CO ligand to create an open coordination site which allows for the binding of a (cyclo)alkane via a sigma complex. Nano-second time-resolved infrared spectroscopy has been key in determining experimental numbers for oxidative addition of C-H bonds to support the ever-growing catalogue of computationally derived data. George and co-workers have been at the forefront of the community, using TR-IR (Time-Resolved Infrared spectroscopy) to show that oxidative addition of (cyclo)alkanes is not necessarily a straightforward endeavour. For example, once coordinated via a sigma complex the C-H bonds around a cycloalkane become chemically inequivalent. This can either increase or decrease the barrier to isomerisation between sigma complexes depending on the size of the ring. This has a particularly interesting effect when considering cycloalkanes with a ring size greater than 8, whereby the isomerisation between sigma complexes becomes more favourable than oxidative addition of the C-H bond. Furthermore, lower barriers to oxidative addition occur when the sigma complex is formed from the interaction of an equatorial C-H bond rather than an axial one. George and co-workers suggest this occurs as a result of lowered steric congestion. However, equatorial bonds are shorter than axial ones which leads to higher barriers of sigma complex formation and is due to the less efficient donation of the electrons to the metal centre^36^.

Some conclusions that can be made regarding the activation of C-H bonds in these systems are that primary C-H bonds are activated more favourably compared to secondary C-H bonds in straight-chain alkanes despite their higher BDE. Secondary C-H bonds directly attached to a CH_3_ group have significantly increased activation barriers. Furthermore, internal CH_2_ groups show higher barriers than those bound to a CH_3_ group. The influence of the spectator ligands, such as Cp and Cp* have a significant effect on the activation barriers of straight-chain alkanes and cycloalkanes. For a full interpretation of the computational and experimental data, the original reports should be consulted as well as the landmark studies by Jones, who showed the correlations between C-H and M-C bond strength in C-H activation reactions^67,68^.

## Sigma bond metathesis (SBM/MAσBM/OATS/OHM/σ-CAM)

**Fig. 6:**
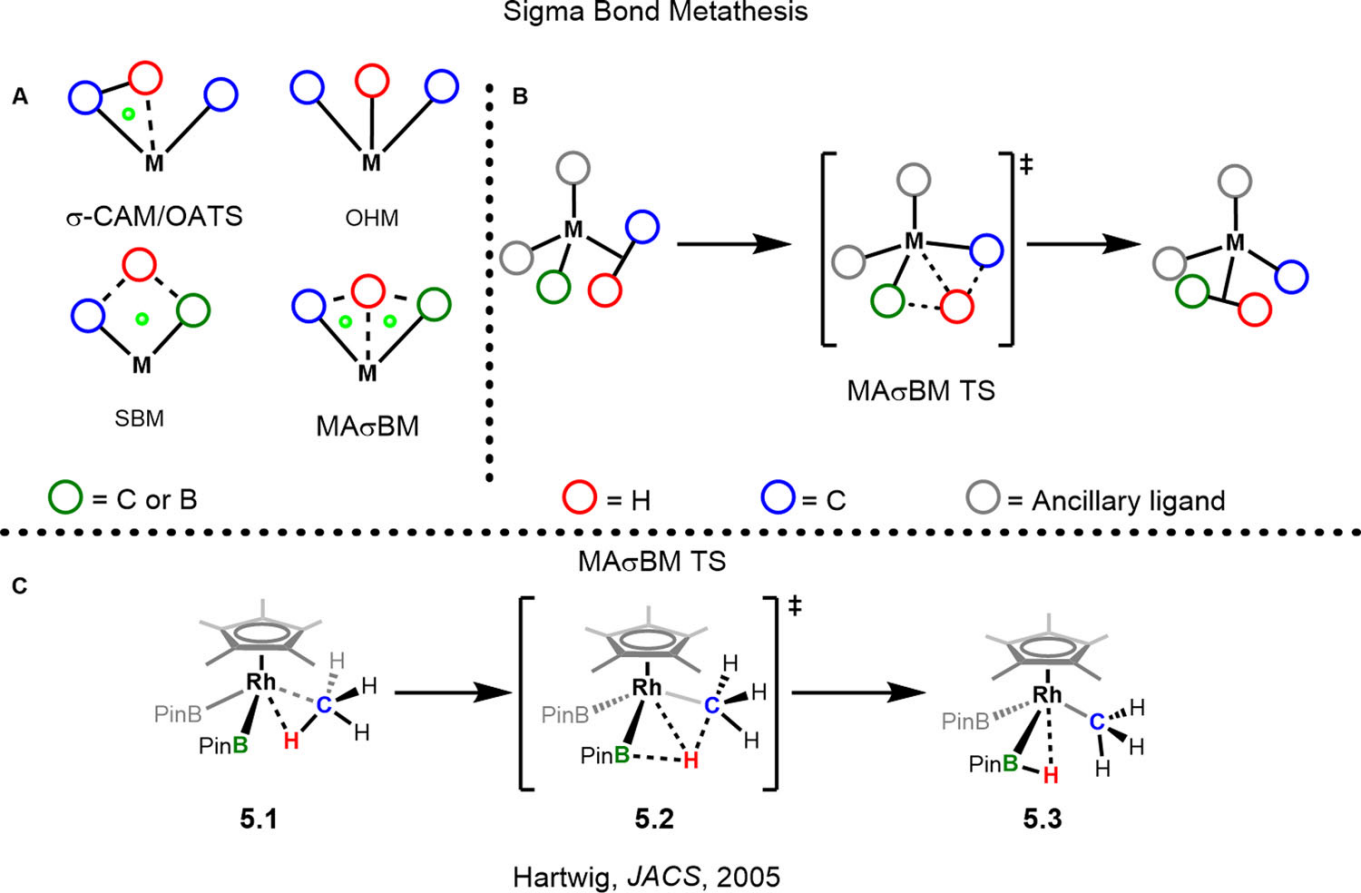
Sigma bond metathesis mechanisms. **A** Transition states encountered during the different sigma bond metathesis mechanisms; green dots represent the ring critical points of the interaction which show the point of minimum electron density in the interaction^76^. **B** Simplified reaction mechanism for MAσBM reaction. **C** Hartwig’s alkane borylation reaction pathway.

Traditionally sigma bond metathesis reactions of hydrocarbons occur between two hydrocarbyl fragments, a hydrocarbyl and metal hydride or a hydrocarbyl and main group element at early transition metals, lanthanoides and actinoides metals^69,70^. Much of the groundbreaking work has been carried out by Watson and Bercaw^71–74^. While early metals are commonly associated with this mechanism, late transition metals have also been found to operate via similar transition states^64^. The simplistic picture of this mechanism is the transfer of a hydrogen atom between hydrocarbyl or hydride units in a four centre transition state (Fig. 6A, SBM). However, the reality is more complicated and several mechanisms exist which can be differentiated from each other based on the geometry of the atoms involved in the transition state. Akin to the ES/CMD/AMLA mechanisms, the sigma bond metathesis mechanisms are fundamentally similar and can be seen as being a part of a continuum. Although a sigma complex is normally required for the activation of a C-H bond prior to the actual cleavage event, the sigma bond metathesis mechanisms do not necessarily have to proceed through one^75^.

The transition states of the sigma bond metathesis reaction path are somewhat convoluted and several groups have found differences in the degree of 'oxidation' of the metal during the transition state (Fig. 6A)^76^. These examples range from having no metal hydrogen interaction (SBM coined by Bercaw), some degree of metal hydrogen interaction, that is metal-assisted sigma bond metathesis (MAσBM coined by Hall and Hartwig)^77,78^, σ-complex assisted metathesis (σ-CAM coined by Perutz)^79,80^ and oxidatively added transition state (OATS coined by Lin)^81^; to a full metal hydride during the transition state, called oxidative hydrogen migration (OHM coined by Oxgaard)^82^. Vastine and Hall provided an excellent in depth breakdown of the transition states of each^76^. C-H activation reactions involving the sigma bond metathesis mechanism are a highly active area of research, with many researchers publishing in depth computational studies of the diverse range of transition states and pathways these reactions proceed through. It is noteworthy to mention that without computational means, the difference between these mechanisms can be difficult to discern with experimental techniques^76^.

While the MAσBM mechanism is a subset of sigma bond metathesis it has its own nuances^79^. Uniquely, it involves a main group element such as boron. Because boron-hydrogen bonds are able to form stable σ-complexes at a variety of transition metals^83^, the transfer of the hydrogen atom from an alkane to the boryl becomes facile as the hydrogen is shuttled between the carbon atom and the boron atom through the sigma complexes of each fragment (Fig. 6, 5.2).

The MAσBM and σ-CAM mechanisms have common features, but meaningful differences. Both mechanisms utilise sigma complexes during the transition state as well as an interaction of the metal to the hydrogen undergoing activation. However, the difference between the two lies in the interaction of the hydrogen atom with the two pendant fragments, which the hydrogen is being transferred to and from. In the case of the σ-CAM mechanism, there is no interaction of the hydrogen atom and the receiving atom in the TS. On the other hand, the MAσBM mechanism does have an interaction between the transferring hydrogen atom and the receiving atom (Fig. 6A, σ-CAM and MAσBM). The MAσBM mechanism is involved in one of the most successful alkane functionalisation reactions developed to date: the borylation of unbranched alkanes which have a multitude of uses, including Suzuki cross-couplings^77,84^.

The Hartwig group has been one of the leaders in the development of alkane borylation chemistry for several decades^84–87^. They followed up on their original reports of stoichiometric and catalytic alkane borylation chemistry with a series of computational and kinetic investigations detailing the mechanism of the process^77,78^. They found that B_2_Pin_2_ (bis(pinacolato)diboron) reacts faster than HBPin in reactions with alkanes and that the process slowed in the presence of added HBPin. The addition of excess HBPin which slowed the rates of borylation, and the inverse order in HBPin, confirmed that reversible dissociation of HBPin to generate a reactive 16e complex precedes the activation of an alkane^77^. However, it must be noted that these results should be considered tentative, as side reactions involving the formation of other rhodium species did occur.

Importantly, in this system the C-H bond cleavage is not rate-determining and, as such, much information regarding the transition state cannot be acquired from experimental data alone. Here, again, computational calculations have proven to be vital in unravelling the smallest of details regarding the mechanism. It was calculated that a MAσBM mechanism is favoured over other mechanisms which has been reported by other groups as well^88^. Furthermore, the empty Bpin p-orbital was shown to be involved in the transition state with regards to either abstraction of the resulting hydride or direct interaction with the C-H bond during cleavage, further supporting the notion of the MAσBM mechanism being operative^78^. This was again supported by a significant degree of B-H bonding in the transition state which helps weaken the C-H bond. More recently in 2016, Sanford and Mindiola independently developed catalytic systems for the borylation of methane utilising similar boryl species^89,90^. Although the sigma bond metathesis mechanism holds some resemblance to the carbene/imido C-H activation mechanisms, and the orbital interactions are similar, the degree of back-bonding for the boryl species is much lower^77^.

Legzdins and co-workers have also shown that tungsten nitrosyl complexes are active for alkane metathesis with longer alkyl chains being ever more readily activated^91^. Increased reactivity was achieved by substituting tungsten for molybdenum^92^. Even more promising is the selectivity tungsten nitrosyl complexes show for primary C-H bonds over C-X bonds in the same molecule. Such selectivity might allow for late-stage functionalisation of complex molecules, particularly those often seen in the pharmaceutical and agrochemical sectors.

## 1,2-addition

**Fig. 7:**
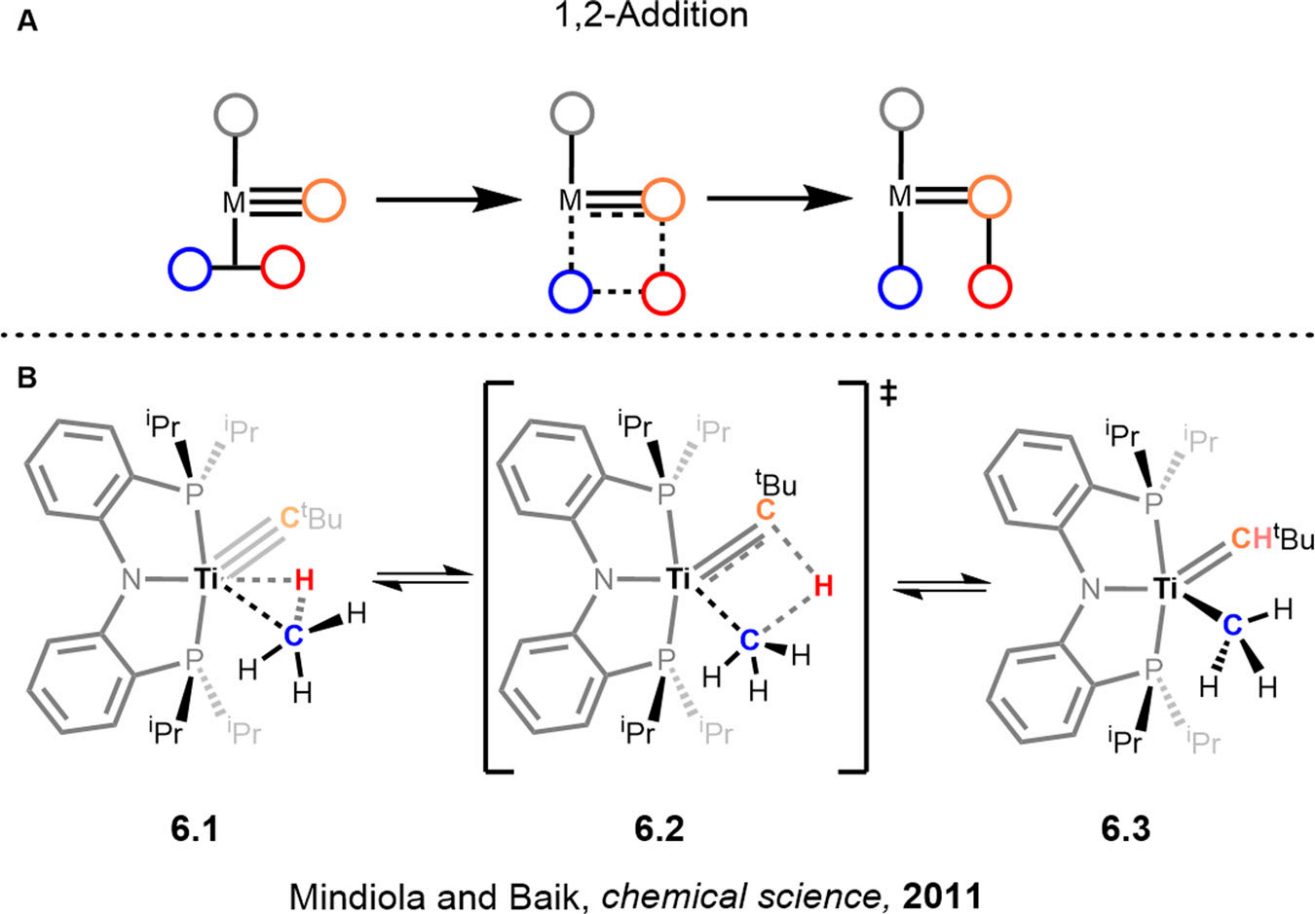
1,2-Addition mechanism. **A** Simplified reaction pathway. **B** Reaction pathway showing the transition state of the 1,2-addition of a C-H bond to a titanium alkylidyne complex. Alkylidene and imido complexes follow the same transition state.

Early metals are also commonly involved in the activation of alkanes through 1,2-addition reactions^93^. For a guide on this topic the tutorial by Wolczanski, who with Bergman first reported the reaction in the late 1980s, is an instructive resource^94^. Initial binding of the alkane in these systems occurs via sigma complex formation to the d_z_^2^ orbital (**6.1**, Fig. 7). The crucial interaction that results in the cleavage and transfer of the hydrogen atom comes from the donation of electron density from the metal-ligand dπ orbital to the R-H σ* orbital resulting in the 4 membered transition state akin to the sigma bond metathesis mechanism (**6.2**, Fig. 7). The activation is then driven forward by the overall bond strength of the resulting M-R bond. This reaction mechanism is active for alkylidene, alkylidyne and imido metal complexes.

Recent advances reported by Mindiola and Baik, studying the reactions of gaseous hydrocarbons with titanium alkylidenes, appear promising for the development of a catalytic process. Much of this work was built upon the titanium alkylidene complexes reported by the Mindiola group since 2005^16,17^. Even these early analogues were successful in activating benzene and Csp^3^ groups of alkyl silanes. However, significant progress was made when in 2011 they reported the activation of methane and ethane^95,96^. Methane was found to be activated at 310 psi at 31 °C across the titanium carbon triple bond with 54% conversion. Almost quantitative conversion was seen if the pressure was increased above 1000 psi. Deuterium labelling studies supported their earlier results that there was no significant KIE in these systems and that the rate-determining step is the formation of the alkylidyne which precedes the C-H activation of methane^95^. Furthermore, CD_4_ activation studies in these systems can rule out other mechanisms if the resulting deuteron ends up fully incorporated in the alkylidene/alkylidyne. This is because the deuteron/proton is shuttled directly to the alkylidene/alkylidyne without the formation of an M-H/D bond that could scramble it into other ligands. This result has also been corroborated for imido complexes^97^.

One of the elegant properties of this system is the ability to study both the experimental kinetic component as well as the computational perspective. The kinetic investigation of the reaction shown in Fig. 7 has revealed the interplay between intermediates and transition states. While the barrier to C-H activation of methane was found to be <24.7 kcal/mol, reversible extrusion of methane from **6.3** (Fig. 7) occurs at a higher barrier of 28.1 kcal/mol. Furthermore, dehydrogenation of methane can also occur but has a minimum requirement >28.1 kcal/mol. Further experiments probing these side reactions showed that, while they are feasible, they also face higher barriers with respect to the main C-H activation pathway^98^.

Ethane C-H activation has, likewise, been studied by Mindiola and Baik, resulting in the first example of  an ethane to ethylene reaction that takes place at room temperature. This reaction has been proposed to occur via a 1,2-addition followed by a beta C-H migration^96^. The conversion of ethane to ethylene is of critical importance due to the expanding usage of polyethylene worldwide. Currently, ethylene is generated via steam cracking. Although industrially viable, the process is energy-intensive and much of the ethane is not converted to ethylene. Furthermore, this mechanism could be looked at favourably for late-stage diversification due to the ability to select Sp3 C-H bonds over Sp2 C-H bonds when the Sp2 bond is less sterically accessible.

## Perspective and outlook

A significant barrier for newcomers to the in depth mechanistic aspects of C-H activation is the lack of clarity around the terminology used in describing mechanistic pathways, and what constitutes a certain type of transition state. Many acronyms exist that describe essentially the same mechanism for a pathway with only slight, nuanced differences between them. In Box 1 we provided not an exhaustive list, but a selection of acronyms that are most associated with their respective mechanism. It can be a challenging task for newcomers to the field to differentiate the nuances of a certain mechanism within the mechanistic continuum based on calculated and experimental data.

With this in mind, given the powerful tools offered by modern computational approaches, increasing collaboration between synthetic and theoretical groups must be undertaken to establish and advance sustainable processes for hydrocarbon functionalisation. Our understanding of how C-H bonds interact with metals and the steric and electronic requirements for promoting C-H bond activation are well established. However, the number of catalytic processes for the functionalisation of alkanes remains scarce.

While this Perspective focused on C-H activation and the mechanisms therein, we would like to end this piece by highlighting some of the work that has been carried out towards C-H bond cleavage which cannot be classified as C-H activation according to the introduced guidelines. Nonetheless, these explorations are equally important in the pursuit of sustainable methods for hydrocarbon functionalisation. These systems include, but are not limited to, enzymatic reactivity^99^, photocatalysis^100–102^, heterogeneous catalysis^103,104^, and main group reactivity^105–107^. The area of C-H activation and functionalisation continues to be of high interest for the chemical community, presumably because of the fundamental and potentially ground-breaking innovations that have yet to be discovered. Taking both, computational and experimental knowledge and combining them into new catalytic methodologies will arguably be a key-step in achieving sustainable homogeneous functionalisation of hydrocarbons. We hope that this brief introduction to the fundamental principles and the associated terminology within this field will guide researchers venturing into the attractive area of C-H bond functionalisation
